# Wide-Band Wide-Beam Circularly-Polarized Slot-Coupled Antenna for Wide-Angle Beam Scanning Arrays

**DOI:** 10.3390/s23031123

**Published:** 2023-01-18

**Authors:** Marco Salucci, Giacomo Oliveri, Mohammad Abdul Hannan, Renzo Azaro, Andrea Massa

**Affiliations:** 1 ELEDIA Research Center, DICAM-Department of Civil, Environmental, and Mechanical Engineering, ELEDIA@UniTN-University of Trento, Via Mesiano 77, 38123 Trento, Italy; 2 ELEDIA Research Unit, CNIT—University of Trento, Via Sommarive 9, 38123 Trento, Italy; 3 ELEDIA Research Center, ELEDIA@UESTC-UESTC, School of Electronic Science and Engineering, University of Electronic Science and Technology of China, Chengdu 611731, China; 4 ELEDIA Research Center, ELEDIA@TSINGHUA-Tsinghua University, 30 Shuangqing Rd, Beijing 100084, China

**Keywords:** wide-beam, wide-band, circular polarization (CP), wide-angle scanning arrays (WASAs), system-by-design (SbD)

## Abstract

The design of a wide-band wide-beam circularly-polarized slot-coupled (WWCS) radiating element for wide-angle scanning arrays (WASAs) is addressed. The WWCS radiator exploits a simple geometry composed of a primary (driven) and a secondary (passive) element to generate wide-beam patterns with rotational symmetry and high polarization purity. The synthesis was carried out by means of a customized version of the System-by-Design (SbD) method to derive a WWCS radiator with circular polarization (CP) and wide-band impedance matching. The results of the numerical assessment, along with a tolerance analysis, confirm that the synthesized WWCS radiating element is a competitive solution for the implementation of large WASAs. More specifically, a representative design working at $f_{0}=2.45$ [GHz] is shown having fractional bandwidth FBW≃15%, half-power beam-width $HPBW\left(f_{0}\right)≃180$ [deg] in all elevation planes, and high polarization purity with broadside axial ratio $AR\left(f_{0}\right)=3.2$ [dB] and cross-polar discrimination $XPD\left(f_{0}\right)=15$ [dB]. Finally, the experimental assessment, carried out on a PCB-manufactured prototype, verifies the wide-band and wide-beam features of the designed WWCS radiator.

## 1. Introduction

In recent decades and within the rapid development of modern wireless systems, there has been a continuously growing interest in beam-scanning antennas [1,2,3]. In such a framework, traditional reflectors provide excellent radiation features (e.g., high gain), but they are bulky and heavy. Moreover, mechanical scanning implies a slow reconfigurability of the main beam direction. Phased antenna arrays are excellent alternative since they guarantee an agile/flexible beam scanning [1,4,5]. As a matter of fact, they have been widely employed in satellite communications, radars, and meteorology [1,4]. Moreover, they will be key technology in next-generation mobile communications systems (i.e., 5G/6G and beyond [2,3]).

Microstrip patch antennas are very popular elementary radiators for phased arrays thanks to several advantages, i.e., they are lightweight, have low profiles, and involve simple/low-cost manufacturing [6,7,8]. However, conventional microstrip-based arrays are usually narrowband [9,10] and they generally exhibit limited scanning capabilities [11]. Since these limitations prevent their use in several applications where a large field-of-view (*FOV*) in a wide-band is required, great efforts have been devoted toward studying innovative solutions for implementing wide-band wide-angle scanning arrays (*WASA*s) [12,13]. In such a framework, wide angle impedance matching (*WAIM*) layers [14,15,16] have been proposed as effective tools to compensate for the mutual coupling among the array elements when steering the beam towards the end-fire for mitigating the decrease of the gain [i.e., the scan loss (*SL*)] due to the mismatch between the antenna and the free-space. Otherwise, pattern-reconfigurable elementary radiators [17,18] have been used to yield large *FOV*s when arranged into arrays thanks to the electronic tilt of the element pattern in the scan direction. However, complex architectures with extra circuits and components are necessary with a higher complexities of the overall system, which may cause a pattern degradation [11,19].

*WASA*s can also be obtained starting from wide-beam radiating elements [10,11,19,20,21,22,23,24,25,26,27,28,29,30,31] according to the different microstrip-based implementations summarized in Table 1. It is worth pointing out that paramount challenges must be properly addressed to design microstrip radiators able to afford radiation patterns with a very large half-power beamwidth (*HPBW*) on the elevation planes and over the wide frequency band (such as, for instance, the design in [11], yielding $HPBW\in \left[156÷360\right]$ [deg] over a fractional bandwidth of FBW=40%—Table 1). Indeed, many state-of-the-art methods for the synthesis of wide-beam antennas broaden the pattern beamwidth either in one plane (i.e., E-plane or H-plane such as, for instance, the design based on “electric walls” [22], which widens the pattern only in the plane containing such parasitic structures—Table 1) or in a narrowband (e.g., FBW=1.2% in [20]—Table 1). Moreover, some of the available designs have rather complex layouts involving quite difficult manufacturing processes, potentially more prone to fabrication tolerances (such as, for instance, the magnetic dipole in [24] and the magnetoelectric dipole with meta-columns loading in [25]—Table 1).

Some interesting approaches implement the wide-beam behavior by adding parasitic elements (e.g., vertical electric walls [22,23], patches [11,19], or rings [20]—Table 1) where additional current components are induced to radiate end-fire patterns that constructively sum with those radiated by the main radiator. Following this guideline, both linearly (*LP*) [11,21,22,23] and circularly (*CP*) polarized [20,25,28] (Table 1) and [11,21,22,23] and circularly (*CP*) polarized [20,25,28] (Table 1) wide-beam radiators were synthesized even though the *CP* ones have several advantages with respect to those with *LP*. For instance, there is an improved immunity to the multi-path distortion, polarization mismatch losses, and Faraday rotation effects caused by the ionosphere in satellite communications [26,27,31]. Thus, *CP* wide-beam radiators are a very promising technological asset for many wireless systems including global positioning and navigation systems (*GPS* and *GNSS*), radars, satellite communications, radio frequency identification, mobile communications, and wireless local area networks [26,27,28].

Accordingly, this paper proposes a novel wide-band wide-beam *CP* slot-coupled (*WWCS*) antenna based on the combination of a *primary* (driven) and a *secondary* (passive) element to generate large-*HPBW* patterns with rotational symmetry and high polarization purity. More specifically, a 3D microstrip layout is obtained by placing a dielectric layer hosting a metallic ring at a proper distance from a circular patch. By properly exciting a *CP* current within such a parasitic element, a torus-shaped pattern with maximum gain on the azimuth plane is radiated, thus triggering an increased end-fire gain which, combining to the broadside radiation of the underlying patch, results in a wide beam along every elevation plane.

Unlike the narrowband design in [20] (having a fractional bandwidth of FBW=1.2%—Table 1), the proposed radiating element is characterized by (*i*) a wide-band impedance matching (i.e., FBW=15%—Table 1), (*ii*) a wider beamwidth (i.e., HPBW≃180 [deg] versus HPBW=131 [deg] of [20] —Table 1) as well as (*iii*) a simpler feeding mechanism for *CP* (i.e., slot-coupling versus probe feeding) [7]. Moreover, unlike the single-element design in [20], the possibility to exploit such an element in a *WASA* is addressed, as well.

Therefore, the main novelties of this work consist of (*a*) the design of a new wide-beam *CP* radiator exploiting an aperture coupling feeding mechanism to significantly widen the impedance bandwidth and overcome spurious radiation, narrowband operation, and more complex manufacturing of probe-fed layouts in the literature [20], (*b*) the formulation of the arising synthesis problem, unlike the parametric *trial-and-error* approach used in [20], as a global optimization enabling a more effective control of the *CP* in the complete radiating semi-sphere and a proper impedance matches within the user-defined wide bands, (*c*) its efficient solution by means of a customized system-by-design (*SbD*) methodology, and (*d*) the wide-band assessment of the suitability of the *WWCS* for implementing large planar *WASA*s, differently from [20] where only the single radiator is considered.

The manuscript is organized as follows. Section 2 describes the layout of the *WWCS* radiator. The *SbD*-based synthesis strategy, which is used for the synthesis of this radiating element, is detailed in Section 3. A representative example, which is concerned with a *LHCP* design, is illustrated in Section 4 to numerically assess, via full-wave (*FW*) simulations along with a tolerance analysis, the effectiveness of the proposed radiator when implementing wide-band *WASA*s. Finally, the experimental assessment of the designed *WWCS* radiator, carried out on a *PCB*-manufactured prototype, is shown in Section 5. Eventually, some conclusions and final remarks are presented in (Section 6).

## 2. *WWCS* Antenna Layout

**Figure 1:**
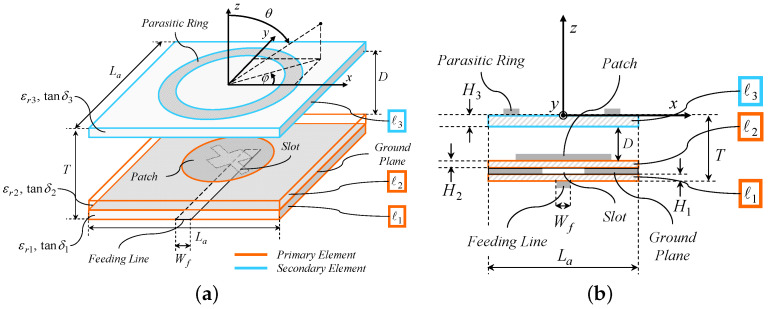
*WWCS Antenna Layout*—Geometry sketch of the proposed *WWCS* radiator: (**a**) *3D* and (**b**) lateral view.

**Figure 2:**
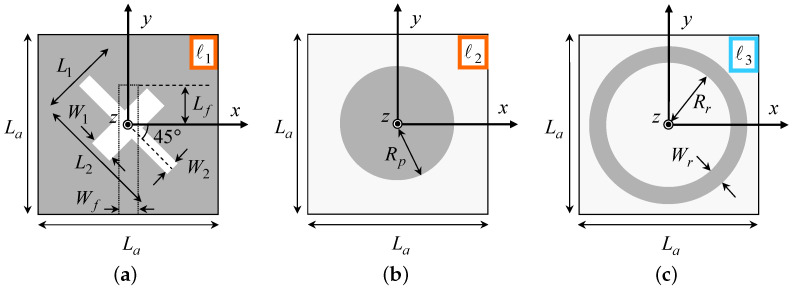
*WWCS Antenna Layout*—top-view geometry sketches of layers (**a**) $\ell_{1}$, (**b**) $\ell_{2}$, and (**c**) $\ell_{3}$.

Figure 1 shows a geometric sketch of the layout of the proposed *WWCS* radiator. The antenna lies on the $\left(x,\;y\right)$ plane and it comprises L=3 square dielectric layers $\ell_{l}$, l=1,…,L of side $L_{a}$. The thickness, the relative permittivity, and the loss tangent of the *l*-th (l=1,…,L) layer are denoted with $H_{l}$, $\varepsilon_{rl}$, and $\tan\delta_{l}$, respectively. The two stacked bottom layers (i.e., $\ell_{1}$ and $\ell_{2}$) are relative to the *primary* antenna element, which consists of a circular microstrip patch of radius $R_{p}$, printed on the layer $\ell_{2}$ [Figure 1 and Figure 2b]. Such a patch is fed with an aperture-coupling mechanism. Towards this end, a cross-shaped slot is etched in the ground plane that separates the layers $\ell_{1}$ and $\ell_{2}$ [Figure 1 and Figure 2a], which is in turn excited with a microstrip feeding line of width $W_{f}$ and characteristic impedance $Z_{0}$. This latter is printed on the bottom face of $\ell_{1}$ [Figure 1and Figure 2a]. To maximize the *EM* coupling, the microstrip line, the slot, and the patch are aligned with respect to the $\left(x,\;y\right)$ plane (Figure 1). Moreover, the feeding line is terminated into an open-circuited stub whose length $L_{f}$ [Figure 2a] is properly tuned so that the standing-wave current, induced within the microstrip, is maximum at the slot barycenter [6].

It is worth pointing out that, even though a multiple-layer etching manufacturing process is required, the adopted aperture feeding enables some advantages with respect to a probe/pin-based choice [6]. For instance, (*a*) is a wide-band impedance matching, (*b*) is a an easier construction, since it avoids the vertical pin that would require additional drilling and soldering processes, and (*c*) is the higher polarization purity and pattern symmetry since the vertical pin would behave as an additional monopole degrading the overall axial ratio (*AR*) and cross-polar discrimination (*XPD*). Moreover, the use of independent substrates for both the circular patch (i.e., $\ell_{2}$) and the feeding line (i.e., $\ell_{1}$) gives the designer more flexibility in selecting the optimum dielectric support for each antenna “building block” with respect to a solution with coplanar edge feeding (either direct or inset-based) [6].

As for the shape of the slot, a 45-degrees rotated cross, with unequal arms of width $W_{1}$ ($W_{2}$) and length $L_{1}$ ($L_{2}$) [Figure 1 and Figure 2a], was adopted to realize the desired circular polarization (*CP*). As a matter of fact, the introduced asymmetry allows one to excite, by injecting a current into the feeding line and exploiting the aperture coupling mechanism, two orthogonal current components having a phase difference of 90 [deg] onto the patch. As a result of the combination of such excited modes, a *CP* current is yielded, which in turns radiates a *CP* field. More specifically, left-hand (*LHCP*) or right-hand (*RHCP*) *CP*s are obtained by simply letting $L_{1}<L_{2}$ or $L_{1}>L_{2}$, respectively [7]. Otherwise, the polarization switching (*LHCP ⇔ RHCP*) could be yielded by simply mirroring the cross aperture with respect to the *y*-axis. Thanks to such a modeling, it is possible to enforce a *CP* by means of a simple design and manufacturing process, since there is no need for two separate orthogonal microstrip lines. Moreover, a simple circular patch can be used by avoiding more complex solutions such as, for instance, a primary element with elliptic-shape (that would imply the tuning of the two semi-axes) or electrically-small perturbations of the external border of the patch (e.g., stubs or notches) to yield an *AR* close to one [7].

The top layer (i.e., $\ell_{3}$) hosts the *secondary* element of the antenna, which is implemented as a metallic ring of inner radius $R_{r}$ and width $W_{r}$ [Figure 1 and Figure 2c]. Such a parasitic element is “activated” by an air coupling mechanism by placing the layer $\ell_{3}$ at a proper distance *D* above the patch (Figure 1). Overall, the total height of the *WWCS* antenna turns out to be (Figure 1)

(1) $$T=D+\sum_{l=1}^{L=3}H_{l}.$$

The secondary passive element shares a geometric rotational symmetry with the primary active one to obtain a high polarization purity and an azimuth-invariant radiation pattern, which is a highly desirable feature for *WASA*s [11]. Indeed, by properly exciting a *CP* current within the parasitic ring [20], a torus-shaped pattern with maximum gain on the azimuth plane [i.e., θ=90 [deg]—Figure 1a)] is radiated. The metallic ring shape is selected to assure that the arising parasitic radiation mode triggers an increased end-fire gain. As a consequence, the combination of the field radiated by the primary element [having maximum gain at broadside, i.e., θ=0 [deg]—Figure 1a] and the secondary radiator generates a wide beam with half-power beamwidth close to $HPBW\left(\varphi \right)=180$ [deg] along every elevation plane $\varphi \in \left[0,\;360\right]$ [deg] [Figure 1a].

## 3. Design Methodology

In order to address in a computationally-effective way the synthesis problem at hand, a customized implementation of the system-by-design (*SbD*) paradigm [32] is exploited and briefly summarized in the following. More specifically, the “*Problem Formulation*” *SbD* functional block [32] is customized to (*i*) define a proper set of geometric descriptors of the *WWCS* layout and (*ii*) formulate a suitable multi-objective cost function accounting for several user-defined requirements on both impedance matching and radiation features. Concerning (*i*), once the characteristics of the substrates (i.e., material/thicknesses) of the layers $\ell_{l}$, l=1,…,L, and the width of the microstrip feeding line $W_{f}$ are determined as detailed in [8] (p. 148, Equation 3.197) to yield the desired characteristic impedance $Z_{0}$ (e.g., $Z_{0}=50$ [Ω]), the set $\underline{\Omega }=\left\{\Omega_{k};\;k=1,\ldots ,K\right\}$ of geometric descriptors (Figure 1 and Figure 2) is defined as $\underline{\Omega }=\left\{R_{p},\;L_{f},\;L_{1},\;W_{1},\;L_{2},\;W_{2},\;D,\;\alpha ,\;\beta \right\}$, α [$\alpha ≜2\times \left(\frac{R_{r}}{L_{a}}\right)$] and β [$\beta ≜\frac{W_{r}}{\left(\frac{L_{a}}{2}-R_{r}\right)}$] being auxiliary parameters (0<α<1; 0<β<1) that avoid the generation of physically-unfeasible geometries for the secondary element by enforcing the constraints $R_{r}<\left(\frac{L_{a}}{2}\right)$ and $W_{r}<\left(\frac{L_{a}}{2}-R_{r}\right)$, respectively [Figure 2c]. The synthesis problem at hand can then be stated as follows:

***WWCS*****Antenna Design Problem**—Determine the optimal setup of the degrees-of-freedoms (*DoF*s), ${\underline{\Omega }}^{\left(opt\right)}$, such that the corresponding *WWCS* radiator (*i*) exhibits a suitable impedance matching within the user-defined wide frequency range $f_{\min}\leq f\leq f_{\max}$, (*ii*) radiates an azimuth-invariant wide-beam pattern suitable for *WASA*s, and (*iii*) implements a *LHCP/RHCP* with high polarization purity within the half-space region $\left(0\leq \theta \leq 90\right)$⋃$\left(0\leq \varphi \leq 360\right)$ [deg] [Figure 1a].

As for (*ii*), because of the conflicting requirements on the bandwidth and the radiation features as well as the non-linear dependence of these latter on $\underline{\Omega }$, the original synthesis problem is recast into a global optimization one, where

(2) $${\underline{\Omega }}^{\left(opt\right)}=\arg\left\{\min_{\underline{\Omega }}\Phi \left(\underline{\Omega }\right)\right\}$$

$\Phi \left(\underline{\Omega }\right)$ being the cost function, which quantifies the mismatch with the synthesis targets, given by

(3) $$\Phi \left(\underline{\Omega }\right)=\sum_{\gamma \in \underline{\Gamma }}w_{\gamma }\Phi_{\gamma }\left(\underline{\Omega }\right)$$

where $\underline{\Gamma }=\left\{S_{11},\;HPBW,\;AR,\;XPD\right\}$, and $w_{\gamma }$ is a real weight associated with the γ-th cost function term $\Phi_{\gamma }\left(\underline{\Omega }\right)$. More in detail, the impedance bandwidth term of the cost function Φ ($\gamma =S_{11}$) is defined as follows

(4) $$\begin{array}{c} \Phi_{S_{11}}\left(\underline{\Omega }\right)=\frac{1}{Q}\sum_{q=1}^{Q}\frac{\left\{\left|S_{11}^{\left(dB\right)}\left(\left.f_{q}\right|\underline{\Omega }\right)\right|-S_{11}^{th}\right\}}{\left|S_{11}^{th}\right|}\times \\ \times H\left\{\left|S_{11}^{\left(dB\right)}\left(\left.f_{q}\right|\underline{\Omega }\right)\right|-S_{11}^{th}\right\} \end{array}$$

where

(5) $$S_{11}^{\left(dB\right)}\left(\left.f_{q}\right|\underline{\Omega }\right)=20\times \log_{10}\left(\frac{Z_{in}\left(\left.f_{q}\right|\underline{\Omega }\right)-Z_{0}}{Z_{in}\left(\left.f_{q}\right|\underline{\Omega }\right)+Z_{0}}\right)$$

is the reflection coefficient at the antenna input port, $Z_{in}\left(\left.f_{q}\right|\underline{\Omega }\right)$ being the input impedance. Moreover, $S_{11}^{th}$ is the desired threshold and $f_{q}$ [$f_{q}≜f_{\min}+\left(q-1\right)\times \frac{\left(f_{\max}-f_{\min}\right)}{\left(Q-1\right)}$] is the *q*-th (q=1,…,Q) frequency sample, *Q* being the number of spectral components analyzed with full-wave (*FW*) simulations. Finally, $H\left\{\;.\;\right\}$ is the Heaviside’s function, equal to $H\left\{\zeta \right\}=1$ if ζ>0 and $H\left\{\zeta \right\}=0$, otherwise. As for the wide-beam features, the *HPBW* cost term (γ=HPBW) is given by

(6) $$\begin{array}{c} \Phi_{HPBW}\left(\underline{\Omega }\right)=\frac{1}{Q\times M}\sum_{q=1}^{Q}\sum_{m=1}^{M}\\ \frac{\left\{HPBW^{th}-HPBW\left(\left.f_{q},\;\varphi_{m}\right|\underline{\Omega }\right)\right\}}{HPBW^{th}}\times \\ \times H\left\{HPBW^{th}-HPBW\left(\left.f_{q},\;\varphi_{m}\right|\underline{\Omega }\right)\right\} \end{array}$$

where $HPBW^{th}$ is the user-defined requirement, while $\varphi_{m}$ ($\varphi_{m}=\left(m-1\right)\times \frac{360}{M}$ [deg]) is the *m*-th (m=1,…,M) azimuth angular sample [Figure 1a], *M* being the number of elevation planes considered for the numerical evaluation of the *HPBW*.

The last two cost function terms in (3) (i.e., γ=AR and γ=XPD) are related to the *CP* and they are defined as follows

(7) $$\begin{array}{c} \Phi_{AR}\left(\underline{\Omega }\right)=\frac{1}{Q\times V\times M}\sum_{q=1}^{Q}\sum_{v=1}^{V}\sum_{m=1}^{M}\\ \frac{\left\{AR^{\left(dB\right)}\left(\left.f_{q},\;\theta_{v},\;\varphi_{m}\right|\underline{\Omega }\right)-AR^{th}\right\}}{AR^{th}}\times \\ \times H\left\{AR^{\left(dB\right)}\left(\left.f_{q},\;\theta_{v},\;\varphi_{m}\right|\underline{\Omega }\right)-AR^{th}\right\} \end{array}$$

and

(8) $$\begin{array}{c} \Phi_{XPD}\left(\underline{\Omega }\right)=\frac{1}{Q\times V\times M}\sum_{q=1}^{Q}\sum_{v=1}^{V}\sum_{m=1}^{M}\\ \frac{\left\{XPD^{th}-XPD^{\left(dB\right)}\left(\left.f_{q},\;\theta_{v},\;\varphi_{m}\right|\underline{\Omega }\right)\right\}}{XPD^{th}}\times \\ \times H\left\{XPD^{th}-XPD^{\left(dB\right)}\left(\left.f_{q},\;\theta_{v},\;\varphi_{m}\right|\underline{\Omega }\right)\right\}. \end{array}$$

In the previous expressions, $\theta_{v}=\left(v-1\right)\times \frac{90}{\left(V-1\right)}$ [deg] is the *v*-th (v=1,…,V) elevation angle [Figure 1a], $AR^{th}$ is the maximum *AR* given by [6]

(9) $$\begin{array}{c} AR^{\left(dB\right)}\left(\left.f_{q},\;\theta_{v},\;\varphi_{m}\right|\underline{\Omega }\right)=\\ =20\times \log_{10}\left(\frac{\left|E_{C}\left(\left.f_{q},\;\theta_{v},\;\varphi_{m}\right|\underline{\Omega }\right)\right|+\left|E_{X}\left(\left.f_{q},\;\theta_{v},\;\varphi_{m}\right|\underline{\Omega }\right)\right|}{\left|E_{C}\left(\left.f_{q},\;\theta_{v},\;\varphi_{m}\right|\underline{\Omega }\right)\right|-\left|E_{X}\left(\left.f_{q},\;\theta_{v},\;\varphi_{m}\right|\underline{\Omega }\right)\right|}\right) \end{array}$$

where the subscripts “C” and “X” denote the co-polar and the cross-polar field components, respectively (i.e., C←LHCP and X←RHCP if a *LHCP* antenna is designed, and vice-versa for *RHCP* operation), equal to

(10) $$\left\{\begin{array}{c} E_{C}\left(\left.f_{q},\;\theta_{v},\;\varphi_{m}\right|\underline{\Omega }\right)=E\left(\left.f_{q},\;\theta_{v},\;\varphi_{m}\right|\underline{\Omega }\right)\cdot {\left({\hat{\rho }}_{C}\right)}^{*}\\ E_{X}\left(\left.f_{q},\;\theta_{v},\;\varphi_{m}\right|\underline{\Omega }\right)=E\left(\left.f_{q},\;\theta_{v},\;\varphi_{m}\right|\underline{\Omega }\right)\cdot {\left({\hat{\rho }}_{X}\right)}^{*}, \end{array}\right.$$

$E\left(\left.f_{q},\;\theta_{v},\;\varphi_{m}\right|\underline{\Omega }\right)=E_{\theta }\left(\left.f_{q},\;\theta_{v},\;\varphi_{m}\right|\underline{\Omega }\right)\hat{\theta }+E_{\varphi }\left(\left.f_{q},\;\theta_{v},\;\varphi_{m}\right|\underline{\Omega }\right)\hat{\varphi }$ being the far-field electric field, $\left(\cdot \right)$ is the dot product, and ${\left(\;.\;\right)}^{*}$ stands for complex conjugate. Moreover, ${\hat{\rho }}_{C}$ and ${\hat{\rho }}_{X}$ are the polarization unit vectors for the two *CP*s [6]

(11) $$\left\{\begin{array}{c} {\hat{\rho }}_{C}=\frac{1}{\sqrt{2}}\left(\hat{\theta }+j\hat{\varphi }\right)\\ {\hat{\rho }}_{X}=\frac{1}{\sqrt{2}}\left(\hat{\theta }-j\hat{\varphi }\right) \end{array}\right..$$

Finally, $XPD^{th}$ is the minimum *XPD* being

(12) $$\begin{array}{c} XPD^{\left(dB\right)}\left(\left.f_{q},\;\theta_{v},\;\varphi_{m}\right|\underline{\Omega }\right)\\ =10\times \log_{10}\left(\frac{G_{C}\left(\left.f_{q},\;\theta_{v},\;\varphi_{m}\right|\underline{\Omega }\right)}{G_{X}\left(\left.f_{q},\;\theta_{v},\;\varphi_{m}\right|\underline{\Omega }\right)}\right) \end{array}$$

where

(13) $$\begin{array}{c} G_{C/X}\left(\left.f_{q},\;\theta_{v},\;\varphi_{m}\right|\underline{\Omega }\right)=\frac{2\pi }{\eta_{0}P_{acc}\left(\left.f_{q}\right|\underline{\Omega }\right)}\\ \times {\left|E_{C/X}\left(\left.f_{q},\;\theta_{v},\;\varphi_{m}\right|\underline{\Omega }\right)\right|}^{2} \end{array}$$

is the gain related to the C/X-th field component, respectively, $\eta_{0}$ is the free-space impedance, while $P_{acc}\left(\left.f_{q}\right|\underline{\Omega }\right)$ is the accepted power at the antenna terminals for a given incident power $P_{inc}$

$$P_{acc}\left(\left.f_{q}\right|\underline{\Omega }\right)=\left(1-10^{\left|S_{11}^{\left(dB\right)}\left(\left.f_{q}\right|\underline{\Omega }\right)\right|/10}\right)\times P_{inc}.$$

The overall *SbD*-driven design work-flow consists of the following procedural steps:

1. *Input phase.* Define the bounds of the target’s operating band, $f_{\min}$ and $f_{\max}$, the required *CP* (i.e., *LHCP* or *RHCP*), and the threshold value for each key performance indicator, ${\underline{\Gamma }}^{th}=\left\{S_{11}^{th},\;HPBW^{th},\;AR^{th},\;XPD^{th}\right\}$. Perform the following operations
  (a) Set $L_{a}=\frac{\lambda_{0}}{2}$, $\lambda_{0}$ being the free-space impedance at the central frequency $f_{0}=\frac{\left(f_{\min}+f_{\max}\right)}{2}$;
  (b) Select from an off-the-shelf data-sheet the material/thickness of the *l*-th (l=1,…,L) layer $\ell_{l}$;
  (c) Compute the width of the feeding line $W_{f}$ to yield the desired characteristic impedance $Z_{0}$ (p. 148, Equation 3.197 [8]);
  (d) Derive an analytic guess, ${\tilde{R}}_{p}$, for the radius of the primary element of the radiator as detailed in [33] (p. 846, Equation 14.69), then set its optimization range $\Omega_{1}^{\left(\min\right)}$ and $\Omega_{1}^{\left(\max\right)}$ as a percentage of ${\tilde{R}}_{p}$, being $\Omega_{1}=R_{p}$;
  (e) Define the optimization bounds of the remaining $\left(K-1\right)$ *DoF*s $\left[\Omega_{k}^{\left(\min\right)},\;\Omega_{k}^{\left(\max\right)}\right]$, k=2,…,K;
2. *Surrogate model (SM) Building.* Use the Latin hypercube sampling (*LHS*) strategy [32] to generate a training set $T_{0}=\left\{\left[{\underline{\Omega }}^{\left(s\right)},\;\Phi \left({\underline{\Omega }}^{\left(s\right)}\right)\right];\;s=1,...,S_{0}\right\}$ of $S_{0}$ training samples, $\Phi \left({\underline{\Omega }}^{\left(s\right)}\right)$ being the cost function associated with the *s*-th ($s=1,...,S_{0}$) input sample ${\underline{\Omega }}^{\left(s\right)}\in \left[{\underline{\Omega }}^{\left(\min\right)},\;{\underline{\Omega }}^{\left(\max\right)}\right]$, computed with a *FW* simulation. Starting from $T_{0}$, build a computationally-fast *SM* of (3) based on the ordinary Kriging (*OK*) learning-by-examples (*LBE*) technique [32,34,35,36,37];
3. *Design Initialization* (i=0)—Define an initial swarm of *P* particles, $P_{0}=\left\{{\underline{\Omega }}_{0}^{\left(p\right)};\;p=1,\ldots ,P\right\}$, with random velocities $V_{0}=\left\{{\underline{\upsilon }}_{0}^{\left(p\right)};\;p=1,\ldots ,P\right\}$;
4. *SbD Design Loop* (i=1,…,I)—Iteratively update the swarm positions and velocities by applying the *PSO-OK/C* updating rules [32], $\left\{P_{i},\;V_{i}\right\}=U\left\{P_{i-1},\;V_{i-1},\;T_{i-1}\right\}$, and leveraging on both the cost function predictions and the associated “reliability estimations” outputted by the *SM.* As for the latter, the training set at the *i*-th (i=1,…,I) iteration, $T_{i}$, of size $S_{i}=\left(S_{0}+i\right)$, comprises progressively-added training samples according to the *SbD* “reinforcement learning” strategy [32] aimed at refining the prediction accuracy within the attraction basin of ${\underline{\Omega }}^{\left(opt\right)}$;
5. *Output Phase*—Output the final setup of the *DoF*s, ${\underline{\Omega }}^{\left(opt\right)}$, whose corresponding layout best fits all user-defined requirements.

## 4. Numerical Assessment

This section is aimed at illustrating the performance of the proposed *WWCS* antenna model. Towards this end, the synthesis of a *LHCP*-polarized radiator working in the $f\in \left[2.3,\;2.6\right]$ [GHz] band (⇒$f_{0}=2.45$ [GHz] and $\lambda_{0}=122.44$ [mm] [20]) was addressed. The Rogers RO4350B substrate was chosen for the L=3 layers ($\varepsilon_{rl}=3.66$, $\tan\delta_{l}=0.004$, l=1,…,L) with thicknesses set to $H_{1}=H_{3}=3.04$ [mm] and $H_{2}=4.56$ [mm]. According to [8], the width of the microstrip feeding line turns out to be $W_{f}=6.65$ [mm] for $Z_{0}=50$ [Ω], while the analytic guess of the patch radius is set to ${\tilde{R}}_{p}=17.46$ [mm] [33]. The *PSO-OK/C* parameters were chosen by following the literature guidelines to yield a time saving of $\Delta t_{sav}=86\%$ with respect to a standard optimization based on a bare integration of the global optimizer and the *FW* simulator to compute the cost function values in correspondence with each trial antenna layout [32].

More specifically, the swarm size, the number of iterations, the social/cognitive acceleration coefficients, the inertial weight, and the initial training size were set to P=9, I=200, $C_{1}=C_{2}=2$, ω=0.4, and $S_{0}=45$, respectively. Moreover, the numerical evaluation of (3) was performed by sampling into Q=3, M=13, and V=4 samples the frequency, the azimuth, and the elevation range, respectively, while letting $S_{11}^{th}=-10$ [dB], $HPBW^{th}=180$ [deg], $AR^{th}=6$ [dB], and $XPD^{th}=15$ [dB]. Finally, the optimization bounds were set to $R_{p}\in \left[0.8,\;1.2\right]\times {\tilde{R}}_{p}$, $L_{f}\in \left[0.1,\;0.9\right]\times \frac{L_{a}}{2}$, $L_{1}=L_{2}$ $\in \left[0.1,\;0.9\right]\times \frac{L_{a}}{2}$, $W_{1}=W_{2}$ $\in \left[0.1,\;0.9\right]\times \frac{L_{a}}{2}$, $D\in \left[0.1,\;1\right]\times \lambda_{0}$, and α=β$\in \left[0.1,\;0.9\right]$.

**Figure 3:**
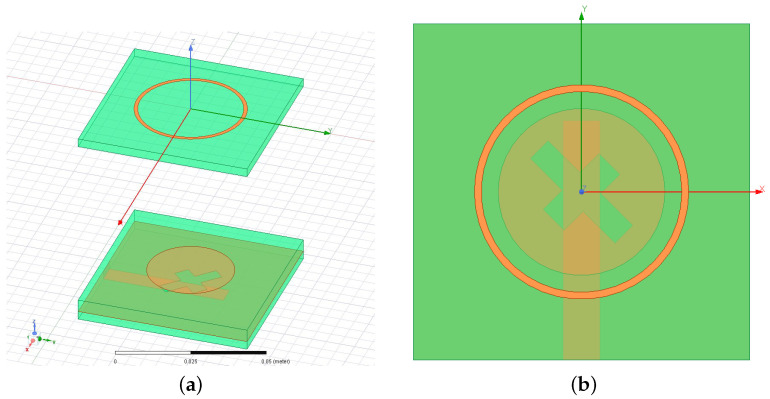
*Numerical assessment* ($\left[f_{\min},\;f_{\max}\right]=\left[2.3,\;2.6\right]$ [GHz])—*SbD*-optimized layout of the designed *WWCS*antenna. (**a**) 3D view and (**b**) top view.

**Figure 4:**
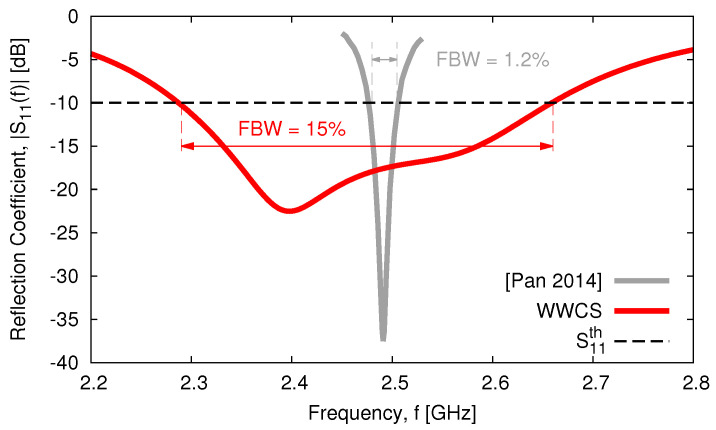
*Numerical assessment* ($\left[f_{\min},\;f_{\max}\right]=\left[2.3,\;2.6\right]$ [GHz])—behavior of the reflection coefficient at the antenna input port, $\left|S_{11}^{\left(dB\right)}\left(f\right)\right|$, and comparison with the literature results in [20].

The geometric descriptors of the *SbD*-optimized layout are reported in Table 2, while the corresponding layout, modeled in the Ansys *HFSS FW* simulator [38] and having an overall height of T=71.98 [mm] (T=0.59 [$\lambda_{0}$] —Table 1), is shown in Figure 3. Going to the analysis of the antenna performance, Figure 4 shows the simulated reflection coefficient at the antenna input port versus the frequency. As it can be observed, such a radiating element fully complies with the requirement since $\left|S_{11}^{\left(dB\right)}\left(\left.f\right|{\underline{\Omega }}^{\left(opt\right)}\right)\right|\leq -11.3$ [dB] for $f\in \left[f_{\min},\;f_{\max}\right]$.In more detail, it turns out that $\left|S_{11}^{\left(dB\right)}\left(\left.f\right|{\underline{\Omega }}^{\left(opt\right)}\right)\right|\leq S_{11}^{th}$ for an even wider frequency interval ($f\in \left[2.29,\;2.66\right]$ [GHz]) by assessing the wide-band behavior of the proposed design with an overall fractional bandwidth of ${\left.FBW\right|}_{WWCS}=15\%$ [39], while, for instance, the state-of-the-art solution in [20] is limited to ${\left.FBW\right|}_{\left[Pan\;2014\right]}=1.2\%$ (Figure 4 and Table 1).

**Figure 5:**
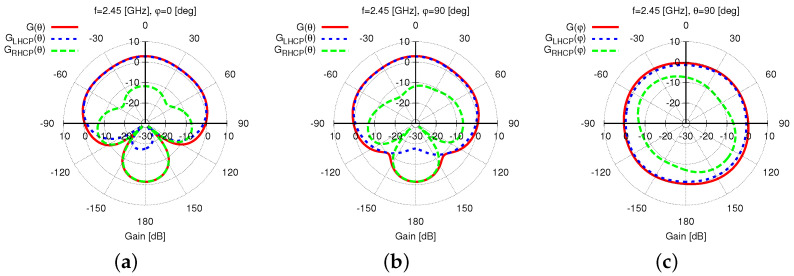
*Numerical Assessment*($\left[f_{\min},\;f_{\max}\right]=\left[2.3,\;2.6\right]$ [GHz], $f=f_{0}=2.45$ [GHz])—Total, $G\left(f_{0},\;\theta ,\;\varphi \right)$, *LHCP*, $G_{LHCP}\left(f_{0},\;\theta ,\;\varphi \right)$, and *RHCP*, $G_{RHCP}\left(f_{0},\;\theta ,\;\varphi \right)$, gain patterns for elevation cuts at (**a**) φ=0 [deg] and (**b**) φ=90 [deg], and (**c**) azimuth cut (θ=90 [deg]).

**Figure 6:**
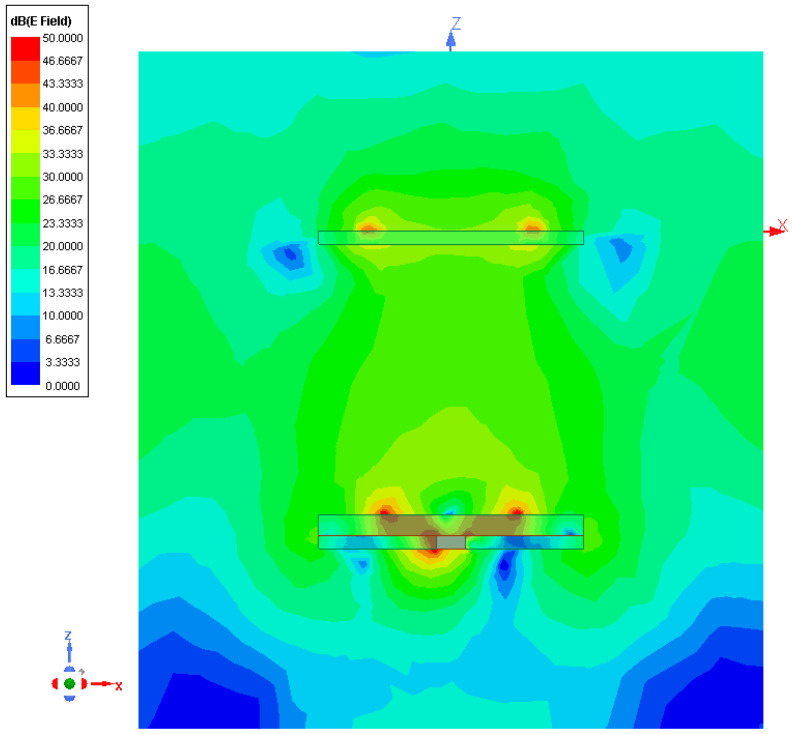
*Numerical assessment* ($\left[f_{\min},\;f_{\max}\right]=\left[2.3,\;2.6\right]$ [GHz], $f=f_{0}=2.45$ [GHz])—2D map of the magnitude of the electric field, $\left|E\left(x,\;z\right)\right|$, in a vertical surface parallel to the $\left(x,\;z\right)$-plane and crossing the center of the *WWCS* antenna.

**Figure 7:**
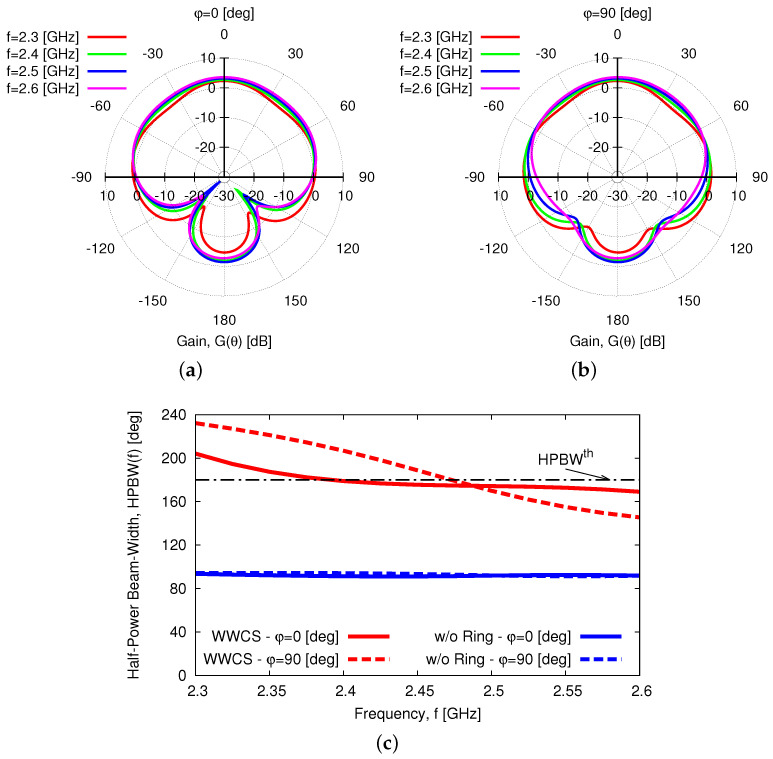
*Numerical Assessment*($\left[f_{\min},\;f_{\max}\right]=\left[2.3,\;2.6\right]$ [GHz])—Plot of (**a**,**b**) the gain pattern at different frequencies in the elevation planes (**a**) φ=0 [deg] and (**b**) φ=90 [deg] and of (**c**) the half-power beamwidth, $HPBW\left(f,\;\varphi \right)$ on both planes, comparing the synthesized *WWCS* radiator with a planar layout obtained by removing the top layer ($\ell_{3}$—Figure 1) hosting the parasitic ring.

As for the radiation features, Figure 5 shows the gain pattern at $f=f_{0}$, $G\left(f_{0},\;\theta ,\;\varphi \right)$, along the two main elevation planes [i.e., φ=0 [deg]—Figure 5a and φ=90 [deg]—Figure 5b] and the azimuth plane [i.e., θ=90 [deg]—Figure 5c]. Since the *HPBW* is equal to $HPBW\left(f_{0},\;\varphi =0\right)=176$ [deg] [Figure 5a] and $HPBW\left(f_{0},\;\varphi =90\right)=189$ [deg] [Figure 5b] on the two vertical cuts, with a good pattern omni-directionality of the whole antenna horizon [Figure 5c], it is reasonable to indicate the proposed antenna such as a wide-beam one suitable for implementing *WASA*s. It is worth noticing that such a feature has been yielded thanks to the constructive combination of the fields radiated by the primary and secondary sources. To better illustrate the *EM* phenomena and interactions, Figure 6 shows the 2D plot of the magnitude of the electric field, $\left|E\left(x,\;z\right)\right|$, on a vertical surface parallel to the $\left(x,\;z\right)$-plane and crossing the barycenter of the antenna. As it can be observed, the air coupling between the bottom (primary) and the top (secondary) element of the radiator at hand guarantees a proper excitation of the parasitic element by enabling the generation of a wide beam in the far-field region. The wide-beam behavior of the synthesized *WWCS* antenna in a wide frequency is “detailed” in Figure 7a,b, where the two elevation gain patterns at O=4 frequencies $f_{o}=f_{\min}+\left(o-1\right)\times \frac{\left(f_{\max}-f_{\min}\right)}{\left(O-1\right)}$, o=1,…,O are given. The remarkable stability, also in frequency, of the wide-beam radiation is pointed out by the plot of the *HPBW* versus the frequency being $HPBW\left(f,\;\varphi =0\right)\geq 169$ [deg] and $HPBW\left(f,\;\varphi =90\right)\geq 146$ [deg] for $f\in \left[f_{\min},\;f_{\max}\right]$ [Figure 7c]. To better understand the advantages of the proposed design, the behavior of the HPBW of a planar radiator obtained by removing the top layer (i.e., $\ell_{3}$—Figure 1) is shown as well [Figure 7c]. It turns out that the presence of the parasitic ring in the synthesized *WWCS* radiator (Figure 1) almost doubles the HPBW at the central frequency, thus verifying its effectiveness in yielding a wide-beam behavior [Figure 7c].

**Figure 8:**
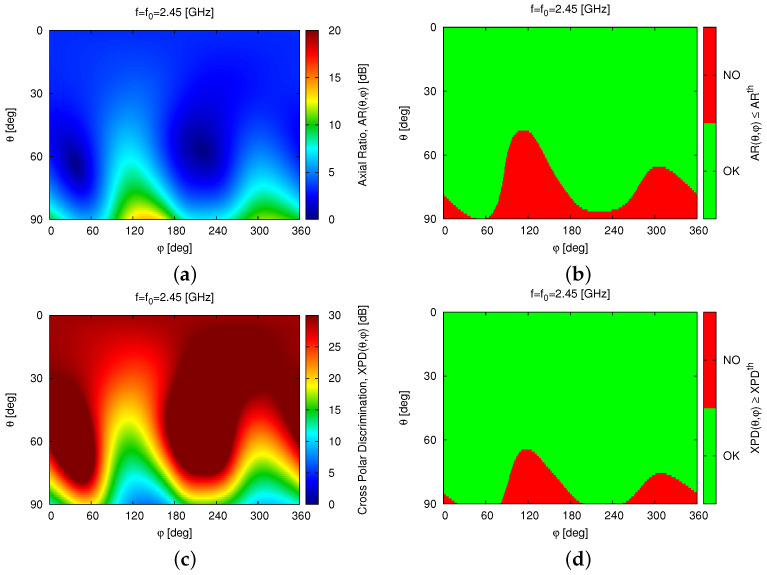
*Numerical Assessment* ($\left[f_{\min},\;f_{\max}\right]=\left[2.3,\;2.6\right]$ [GHz], $f=f_{0}=2.45$ [GHz])—Absolute (**a**,**c**) and thresholded (**b**,**d**) 2D maps of the simulated (**a**,**b**) axial ratio, $AR\left(f_{0},\;\theta ,\;\varphi \right)$, and (**c**,**d**) cross-polar discrimination, $XPD\left(f_{0},\;\theta ,\;\varphi \right)$, in the complete upper half-space of the *WWCS* antenna.

The optimized *WWCS* layout exhibits the desired *LHCP* operation and is pointed out by both the co-polar, $G_{LHCP}\left(f_{0},\;\theta ,\;\varphi \right)$, and the cross-polar, $G_{RHCP}\left(f_{0},\;\theta ,\;\varphi \right)$, gain patterns in Figure 5, where it can be clearly observed that $G\left(f_{0},\;\theta ,\;\varphi \right)\approx G_{LHCP}\left(f_{0},\;\theta ,\;\varphi \right)$ and $G_{LHCP}\left(f_{0},\;\theta ,\;\varphi \right)\gg G_{RHCP}\left(f_{0},\;\theta ,\;\varphi \right)$ for 0≤θ≤90 [deg] with broadside *AR* and *XPD* equal to $AR\left(f_{0},\;\theta =0,\;\varphi =0\right)=3.2$ [dB] and $XPD\left(f_{0},\;\theta =0,\;\varphi =0\right)=15$ [dB], respectively. Moreover, such a good polarization purity is kept almost unaltered in the complete radiating upper semi-sphere with the exception of the elevation angles close to the antenna end-fire as illustrated by the 2D maps of $AR\left(f_{0},\;\theta ,\;\varphi \right)$ [Figure 8a] and $XPD\left(f_{0},\;\theta ,\;\varphi \right)$ [Figure 8c] as well as by the corresponding thresholded pictures aimed at highlighting the fulfilment of the design requirements [Figure 8b,d]. It is worth remarking that the slight degradation of both *AR* and *XPD* appears only in the most challenging region (i.e., θ≃90 [deg]) and is possibly due to the spurious radiation by the slot along the directions of its major arms (i.e., φ=135 [deg] and φ=315 [deg]—Figure 8 and Figure 3).

**Figure 9:**
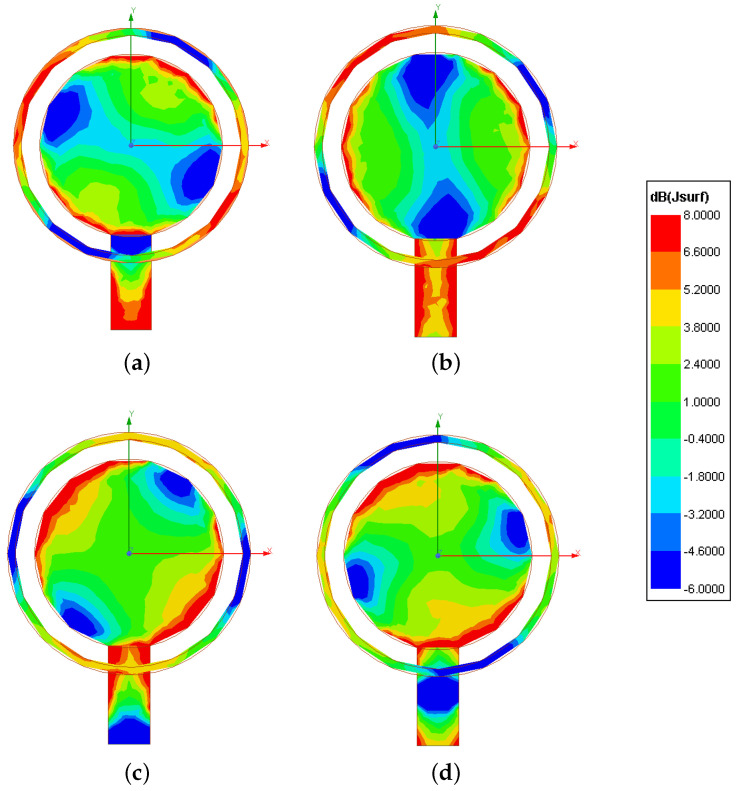
*Numerical Assessment* ($\left[f_{\min},\;f_{\max}\right]=\left[2.3,\;2.6\right]$ [GHz], $f=f_{0}=2.45$ [GHz])—Plot of the instantaneous surface current density magnitude, $\left|J_{surf}\left(x,\;y;\;t\right)\right|$, at the time instants (**a**) t=0 [sec], (**b**) $t=\frac{T_{0}}{8}$ [sec], (**c**) $t=\frac{T_{0}}{4}$ [sec], and (**d**) $t=\frac{3}{8}T_{0}$ [sec].

**Figure 10:**
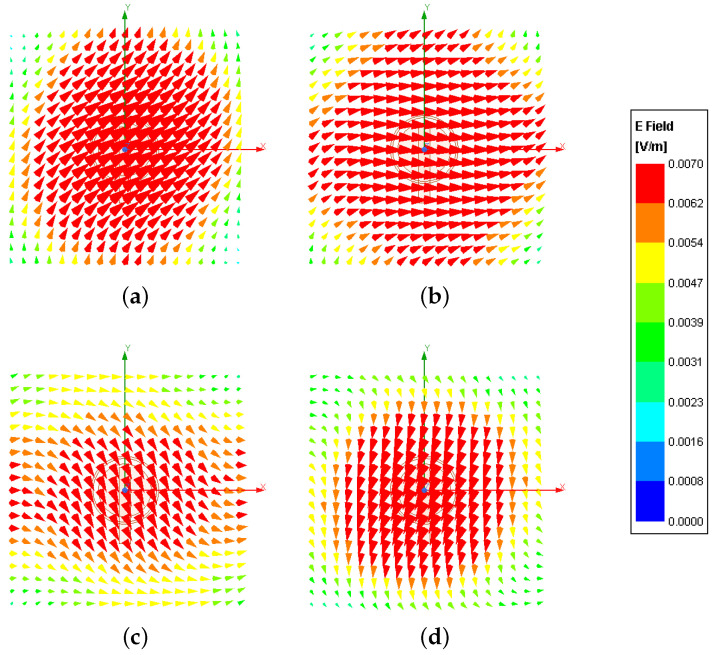
*Numerical Assessment*($\left[f_{\min},\;f_{\max}\right]=\left[2.3,\;2.6\right]$ [GHz], $f=f_{0}=2.45$ [GHz])—Plot of the vectorial electric field at $z=10\lambda_{0}$, $E\left(x,\;y;\;t\right)$, at the time instants (**a**) t=0 [sec], (**b**) $t=\frac{T_{0}}{8}$ [sec], (**c**) $t=\frac{T_{0}}{4}$ [sec], and (**d**) $t=\frac{3}{8}T_{0}$ [sec].

In order to assess the excitation of a *LHCP*, the plot of the magnitude of the instantaneous surface current density, $\left|J_{surf}\left(x,\;y;\;t\right)\right|$, is reported in Figure 9 at four consecutive instants [i.e., t=0 [sec]—Figure 9a; $t=\frac{T_{0}}{8}$ [sec]—Figure 9b; $t=\frac{T_{0}}{4}$ [sec]—Figure 9c; $t=\frac{3}{8}T_{0}$ [sec]—Figure 9d $T_{0}$ being the period at $f_{0}$, $T_{0}=\frac{1}{f_{0}}$]. One can observe that the fundamental mode $TM_{11}$ is properly excited on the circular patch [33] and there is a clock-wise rotation of the corresponding surface current distribution (Figure 9). The vector plot of the electric field distribution at a quota of $z=10\lambda_{0}$, $E\left(x,\;y;\;t\right)$, shown in Figure 10 for the same instants, further verifies the desired *CP* of the radiated wave, which evolves in time according to a *LHCP*.

**Figure 11:**
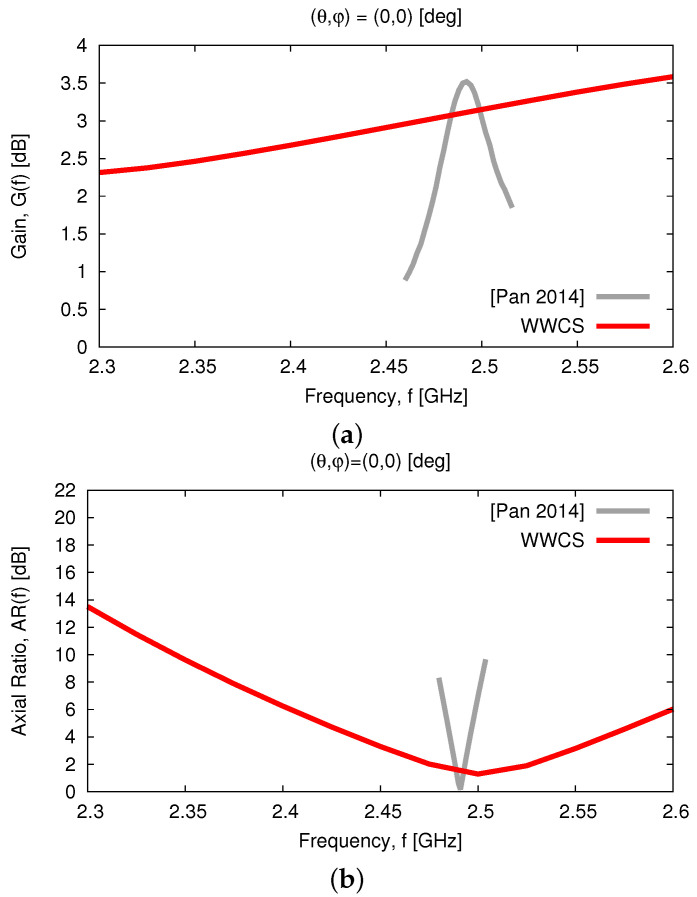
*Numerical Assessment* ($\left[f_{\min},\;f_{\max}\right]=\left[2.3,\;2.6\right]$ [GHz])—Behavior of the (**a**) broadside gain, $G\left(f,\;\theta =0,\;\varphi =0\right)$, and (**b**) axial ratio, $AR\left(f,\;\theta =0,\;\varphi =0\right)$, as compared to the literature result in [20].

For comparison purposes, Figure 11 plots the broadside gain $G\left(f,\;\theta =0,\;\varphi =0\right)$ [Figure 11a] and the *AR* $AR\left(f,\;\theta =0,\;\varphi =0\right)$ [Figure 11b] within the band of interest of the proposed *WWCS* model and of the design in [20]. It turns out that the synthesized radiator exhibits a good *AR* performance, especially within the band $f\in \left[2.4,\;2.6\right]$ [GHz] where ${\left.AR\right|}_{WWCS}\leq 6$ [dB] [40], that results in an *AR* bandwidth (*ARBW*) equal to ${\left.ARBW\right|}_{WWCS}^{6\;dB}=8\%$, while ${\left.ARBW\right|}_{\left[Pan\;2014\right]}^{6\;dB}=0.8\%$ [i.e., an improvement by $\frac{{\left.ARBW\right|}_{WWCS}^{6\;dB}}{{\left.ARBW\right|}_{\left[Pan\;2014\right]}^{6\;dB}}=10$ times—Figure 11b]. A similar outcome is yielded for the 3 [dB]-bandwidth, as well, since ${\left.ARBW\right|}_{WWCS}^{3\;dB}=3.6\%$ and ${\left.ARBW\right|}_{\left[Pan\;2014\right]}^{3\;dB}=0.3\%$ [i.e., an improvement by $\frac{{\left.ARBW\right|}_{WWCS}^{3\;dB}}{{\left.ARBW\right|}_{\left[Pan\;2014\right]}^{3\;dB}}=12$ times—Figure 11b].

**Figure 12:**
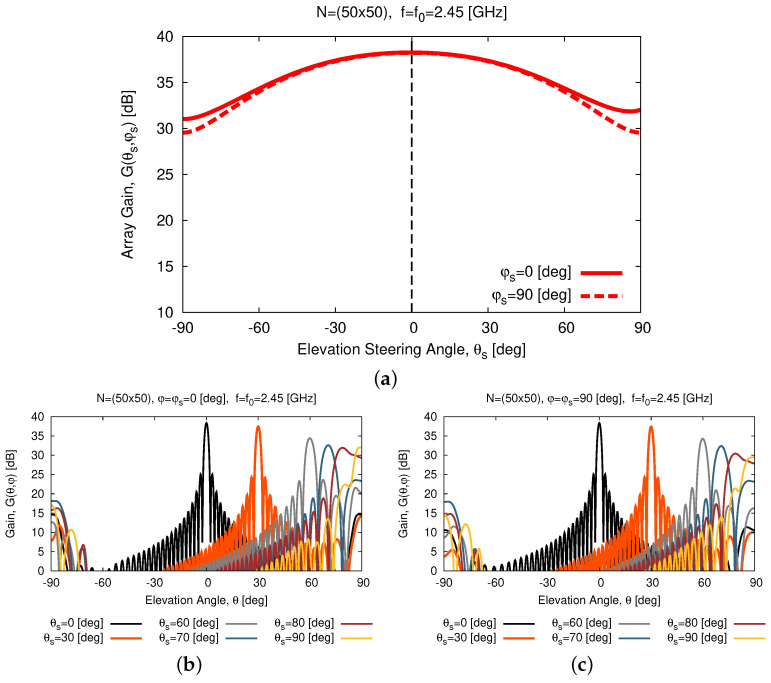
*Numerical Assessment* ($\left[f_{\min},\;f_{\max}\right]=\left[2.3,\;2.6\right]$ [GHz], $f=f_{0}=2.45$ [GHz]—$N=\left(50\times 50\right)$) —Plot of (**a**) the array gain in the angular steering direction, ($\theta_{s}$, $\varphi_{s}$), when scanning the beam towards $\theta_{s}\in \left[-90,\;90\right]$ [deg] and $\varphi_{s}=0$ [deg] or $\varphi_{s}=90$ [deg] along with the corresponding far-field patterns at (**b**) $\varphi_{s}=0$ [deg] and (**c**) $\varphi_{s}=90$ [deg].

Finally, the suitability of the *WWCS* radiator as elementary building block of circularly-polarized wide-band *WASA*s was assessed. Towards this end, the radiation features of a large planar uniform phased array, comprising $N=\left(50\times 50\right)$ *WWCS* identical elementary radiators, were studied. To account for the mutual coupling in this large aperture, a periodic model was simulated in *HFSS*. Figure 12a) describes the behavior of the array gain along the angular steering direction, $\left(\theta_{s},\;\varphi_{s}\right)$, when setting uniform phase shifts and isophoric excitations to scan the beam in the range $\theta_{s}\in \left[-90,\;90\right]$ [deg] in both vertical planes (i.e., $\varphi_{s}=0$ [deg] and $\varphi_{s}=90$ [deg]).To avoid the insurgence of grating lobes for close-to-endfire operation, an inter-element spacing of $d=d_{x}=d_{y}=0.49$ [$\lambda_{0}$] was chosen by slightly shrinking the side of the *WWCS* antenna to $L_{a}=d$.

**Figure 13:**
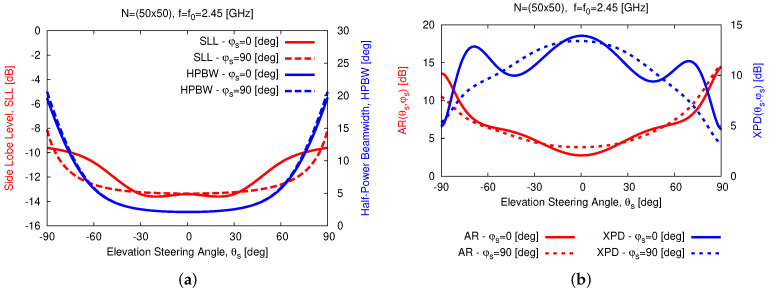
*Numerical Assessment* ($\left[f_{\min},\;f_{\max}\right]=\left[2.3,\;2.6\right]$ [GHz], $f=f_{0}=2.45$ [GHz]—$N=\left(50\times 50\right)$) —Plot of the array (**a**) *SLL*/*HPBW* and (**b**) *AR*/*XPD* when scanning the beam towards $\theta_{s}\in \left[-90,\;90\right]$ [deg] and $\varphi_{s}=0$ [deg] or $\varphi_{s}=90$ [deg], respectively.

Thanks to the wide-beam nature of the *WWCS* elementary radiator, the array exhibits good scan loss (*SL*) performance [$SL\left(\varphi_{s}\right)=G\left(0,\;\varphi_{s}\right)-G\left(90,\;\varphi_{s}\right)$], as illustrated by the patterns at different values of the scan angle $\theta_{s}$ for both $\varphi_{s}=0$ [deg] [Figure 12b] and $\varphi_{s}=90$ [deg] [Figure 12c]. The *SL* on both elevation planes is always smaller than 8.5 [dB] at the central frequency [Figure 12a]. Moreover, it is worth noticing that there is a good stability of the sidelobe level (*SLL*) when scanning the beam on both planes [i.e., −13.6≤SLL≤−8.2 [dB]—Figure 13a].

**Figure 14:**
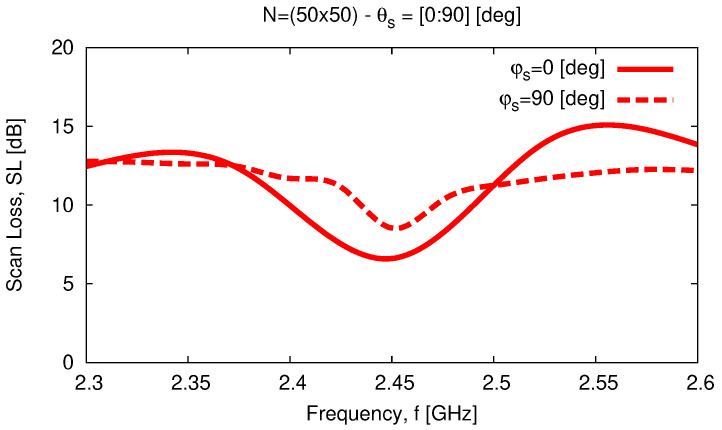
*Numerical Assessment* ($\left[f_{\min},\;f_{\max}\right]=\left[2.3,\;2.6\right]$ [GHz]—$N=\left(50\times 50\right)$)—*SL* performance versus frequency when steering the beam towards $\theta_{s}\in \left[0,\;90\right]$ [deg] and $\varphi_{s}=0$ [deg] or $\varphi_{s}=90$ [deg].

Similar outcomes can be inferred for the array *HPBW* as well, since 2≤HPBW≤6 [deg] for $\left|\theta_{s}\right|\leq 70$ [deg], while it rapidly increases outside such an angular interval because of the beam broadening effect [33] [i.e., HPBW≃20 [deg] for $\theta_{s}=90$ [deg]—Figure 13a and [Figure 12b,c]. Moreover, a good polarization purity is yielded in the whole scan range as indicated by the plot of both the *AR* and the *XPD* along the angular steering direction, since $AR\left(\theta_{s},\;\varphi_{s}\right)\leq 14.6$ [dB] and $XPD\left(\theta_{s},\;\varphi_{s}\right)\geq 3.3$ [dB] when $\left|\theta_{s}\right|\leq 90$ [deg] and $\varphi_{s}=\left\{0;\;90\right\}$ [deg] [Figure 13b]. Finally, let us note that the *WASA* properties of the array are confirmed within the frequency range of interest (Figure 14) where $6.5\leq SL\left(f\right)\leq 15.1$ [dB] for $f_{\min}\leq f\leq f_{\max}$ in both elevation planes.

**Figure 15:**
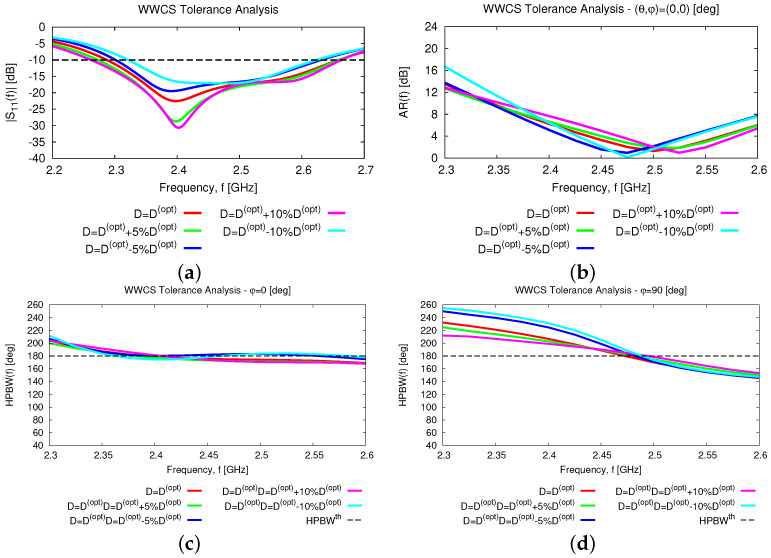
*Numerical Assessment* ($\left[f_{\min},\;f_{\max}\right]=\left[2.3,\;2.6\right]$ [GHz])—Behavior of the (**a**) reflection coefficient, $\left|S_{11}^{\left(dB\right)}\left(f\right)\right|$, (**b**) axial ratio, $AR\left(f,\;\theta =0,\;\varphi =0\right)$, and $HPBW\left(f\right)$ along the (**c**) φ=0 [deg] and (**d**) φ=90 [deg] elevation planes of the *WWCS* radiating element when the height of the parasitic element, *D*, is affected by manufacturing deviations of ±5% and ±10% from the nominal value $D^{\left(opt\right)}$(Table 2).

**Figure 16:**
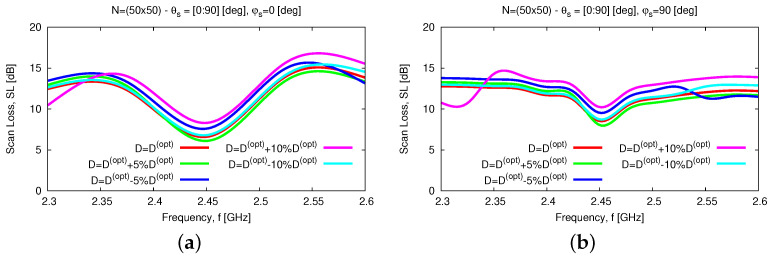
*Numerical Assessment* ($\left[f_{\min},\;f_{\max}\right]=\left[2.3,\;2.6\right]$ [GHz]—$N=\left(50\times 50\right)$)—*SL* performance versus frequency when steering the beam towards $\theta_{s}\in \left[0,\;90\right]$ [deg] and (**a**) $\varphi_{s}=0$ [deg] or (**b**) $\varphi_{s}=90$ [deg] and assuming that the height of the parasitic element, *D*, is affected by manufacturing deviations of ±5% and ±10% from the nominal value $D^{\left(opt\right)}$(Table 2).

In addtion to the numerical assessment, a tolerance analysis has been carried out to give the interested reader some insights into the reliability and robustness on the fabrication tolerances of the proposed antenna layout both stand-alone and within an array arrangement. First, the height of the parasitic element, *D*, has been supposed to deviate of ±5% and ±10% from the nominal value $D^{\left(opt\right)}$(Tab. II) because of some manufacturing tolerances. Figure 15 summarizes the results of the tolerance analysis versus the frequency for the input reflection coefficient [Figure 15a], the broadside *AR* [Figure 15b], and the *HPBW* along the φ=0 [deg] [Figure 15c] and the φ=90 [deg] [Figure 15d] planes. As it can be inferred, the proposed antenna layout turns out to be quite robust. More precisely, the wide-band [Figure 15a] and the wide-beam [Figure 15c,d] characteristics of the *WWCS* radiator are confirmed regardless of the non-negligible fabrication tolerances on *D*, the fractional bandwidth being equal to FBW=12.1% in the worst case [i.e., $D=D^{\left(opt\right)}-10\%D^{\left(opt\right)}$—Figure 15a]. As a consequence, the scan loss value of the array, $SL\left(f\right)$, within the working frequency range, $f_{\min}\leq f\leq f_{\max}$, is quite stable in both elevation planes (Figure 16), as well.

**Figure 17:**
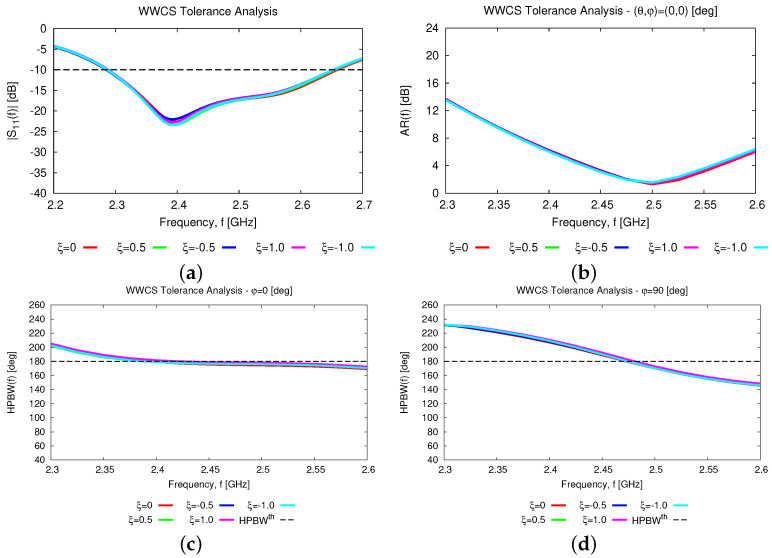
*Numerical Assessment* ($\left[f_{\min},\;f_{\max}\right]=\left[2.3,\;2.6\right]$ [GHz])—Behavior of the (**a**) reflection coefficient, $\left|S_{11}^{\left(dB\right)}\left(f\right)\right|$, (**b**) axial ratio, $AR\left(f,\;\theta =0,\;\varphi =0\right)$, and $HPBW\left(f\right)$ along the (**c**) φ=0 [deg] and (**d**) φ=90 [deg] elevation planes of the *WWCS* radiating element for different settings of the manufacturing tolerance ξ on the feeding line ($W_{f}$) and the parasitic ring ($W_{r}$) widths.

**Figure 18:**
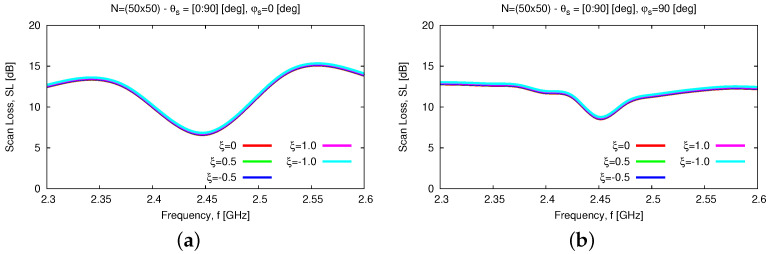
*Numerical Assessment* ($\left[f_{\min},\;f_{\max}\right]=\left[2.3,\;2.6\right]$ [GHz]—$N=\left(50\times 50\right)$)—*SL* performance versus frequency when steering the beam towards $\theta_{s}\in \left[0,\;90\right]$ [deg] and (**a**) $\varphi_{s}=0$ [deg] or (**b**) $\varphi_{s}=90$ [deg] for different settings of the manufacturing tolerance ξ on the feeding line ($W_{f}$) and the parasitic ring ($W_{r}$) widths.

Similar conclusions can be drawn when taking into account fabrication tolerances on the width of the feeding line, $W_{f}$, and the parasitic ring, $W_{r}$, which have been varied according to the following rule: $W_{f/r}=W_{f/r}^{\left(opt\right)}+\left(\xi \times \nu \right)$, ν=0.35 [μm] being the metallization thickness and $\xi \in \left\{-1.0;-0.5;\;+0.5;\;+1.0\right\}$. The effects of such deviations are almost negligible on the features of both the elementary radiator and the array as one can derive from the analysis of the plots in Figure 17 and in Figure 18, respectively.

## 5. Experimental Assessment

**Figure 19:**
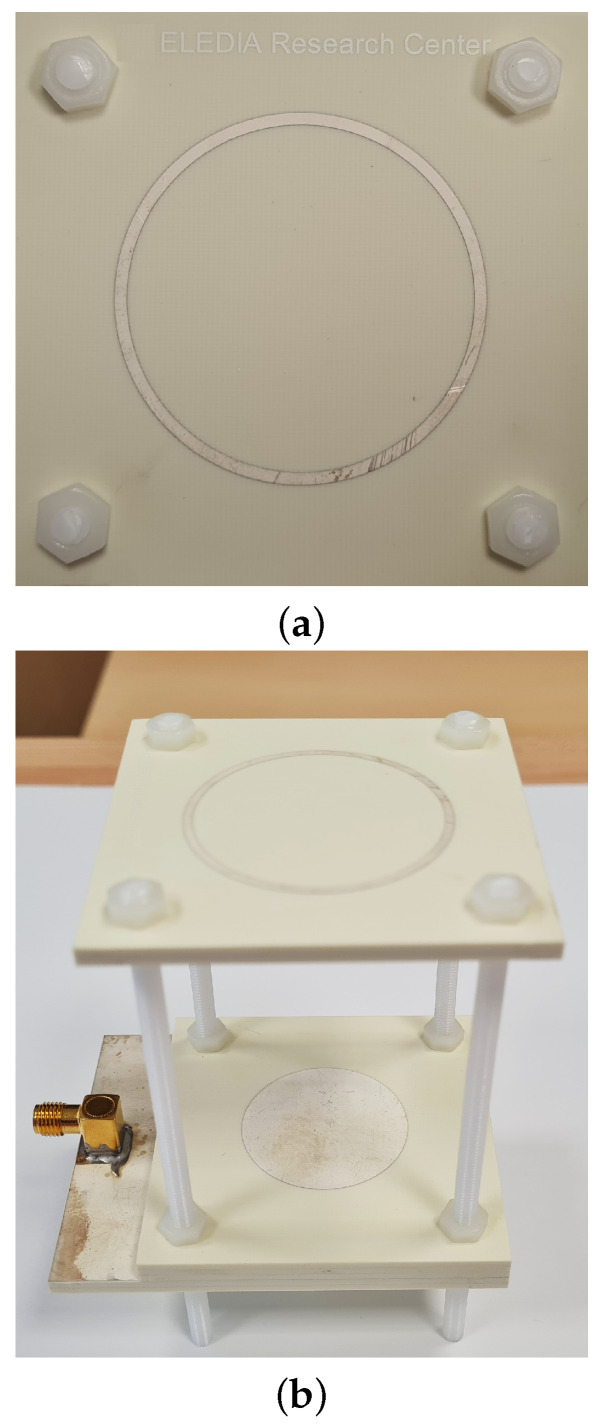
*Experimental Assessment*—Picture of the fabricated prototype: (**a**) top and (**b**) lateral views.

**Figure 20:**
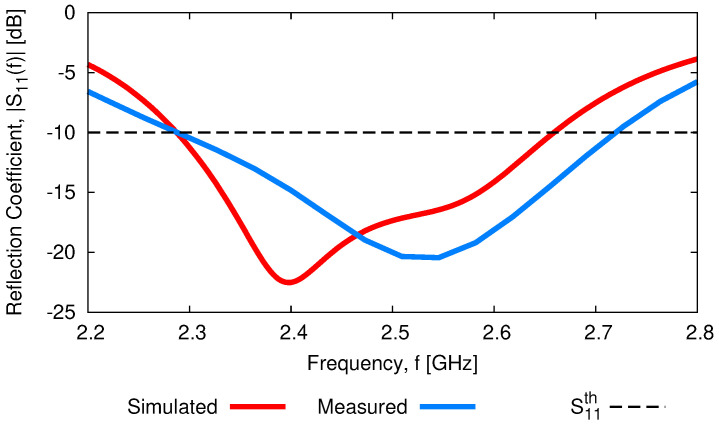
*Experimental Assessment*—Comparison between the simulated and measured reflection coefficients at the antenna input port.

The experimental validation of the performance of the designed *WWCS* has been carried out next (Figure 19). In order to exploit available off-the-shelf RO4350B *PCB* boards, two (layers $\ell_{2}$ and $\ell_{3}$—Figure 1) and three (layer $\ell_{1}$—Figure 1) substrates of thickness h=1.52 [mm] have been stacked to realize the different layers of the antenna. The overall structure has been assembled using four nylon *M4* threaded rods and sixteen nylon bolts, stacking together the *PCB*s and placing the parasitic ring at distance $D=D^{\left(opt\right)}$ from the driven patch (Figure 19). An *RS 759-5252 SMA* connector has been used to feed the antenna prototype. Figure 20 shows the measured reflection coefficient at the input port of the antenna under test (*AUT*) by employing a properly calibrated scalar network analyzer Rohde & Schwarz ZVH4 (100 [kHz]—3.6 [GHz]). As it can be seen, the fabricated *WWCS* exhibits a suitable impedance matching in the complete target band, being ${\left|S_{11}^{\left(dB\right)}\left(f=f_{\min}\right)\right|}_{meas}=-10$ [dB] and ${\left|S_{11}^{\left(dB\right)}\left(f=f_{\max}\right)\right|}_{meas}=-17.8$ [dB]. Moreover, a slightly larger bandwidth has been observed with respect to the *HFSS* simulation (i.e., ${\left.FBW\right|}_{meas}=16.7\%$—Figure 20).

**Figure 21:**
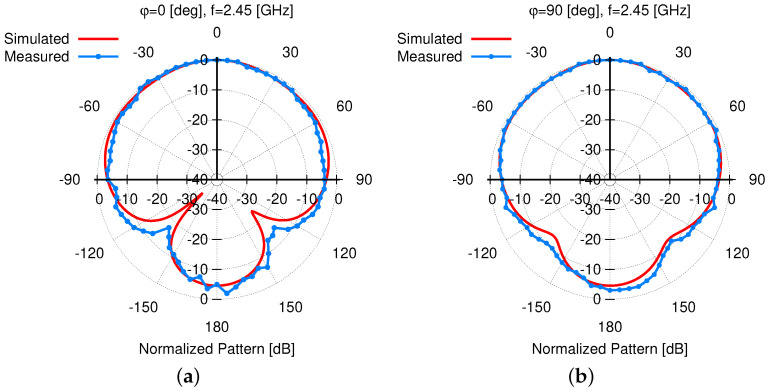
*Experimental Assessment* —Comparison between the simulated and measured patterns at $f=f_{0}=2.45$ [GHz] along the elevation cuts (**a**) φ=0 [deg] and (**b**) φ=90 [deg].

As for the radiation features of the fabricated antenna, the far field patterns have been measured inside an anechoic chamber having dimensions 9×6×6 [$m^{3}$]. The *AUT* has been placed on a remotely controlled rotating frame and the electric field has been measured by means of a circularly polarized probe connected to a signal analyzer, both placed on a dielectric mast at a distance of 3 [m] from the *AUT*. In order to avoid field perturbations due to cablings, the *AUT* has been connected with a short coaxial cable to a small transmitter able to generate a constant amplitude and frequency signal at $f=f_{0}=2.45$ [GHz]. The transmitter has been placed just behind the layer $\ell_{1}$ of the *AUT*. Similarly, the presence of a long coaxial cable connected to the field probe has been avoided thanks to the use of the PMM 9060 EMI Receiver/Signal Analyzer (30 [MHz]–6 [GHz]) that can be remotely controlled by means of a fiber optic link. A good matching between the simulated and measured gain pattern has been obtained. As a matter of fact, both pattern cuts along the φ=0 [deg] [Figure 20a] and the φ=90 [deg] [Figure 21b] elevation planes closely match the outcomes of the numerical assessment. Moreover, it turns out that the measured *HPBW* verifies the wide-beam behavior of the radiator on both planes, being ${\left.HPBW\left(f_{0},\;\varphi =0\right)\right|}_{meas}=151$ [deg] [Figure 21a] and ${\left.HPBW\left(f_{0},\;\varphi =90\right)\right|}_{meas}=172$ [deg] [Figure 21b], respectively. Finally, the measured gain, *AR*, and *XPD* are equal to ${\left.G\left(f_{0},\;\theta =0,\;\varphi =0\right)\right|}_{meas}=2.8$ [dB], ${\left.AR\left(f_{0},\;\theta =0,\;\varphi =0\right)\right|}_{meas}=3.3$ [dB], and ${\left.XPD\left(f_{0},\;\theta =0,\;\varphi =0\right)\right|}_{meas}=14.8$ [dB], respectively, thus verifying a good matching with the simulated values.

## 6. Conclusions

The design of a novel wide-band wide-beam circularly-polarized elementary radiator has been proposed for *WASA*s. Such a *WWCS* structure leverages on a cross-shaped aperture-coupling feeding mechanism to achieve wide-band *LHCP/RHCP* operation using a simple circular patch and a single microstrip line. Moreover, it takes advantage of the air coupling between the primary and secondary *EM* sources to realize rotational-symmetric patterns with large elevation *HPBW*s and high polarization purity in the complete upper semi-sphere. The computationally-efficient synthesis of the layout of the *WWCS* antenna, which supports the desired *CP* operation, has been carried out with a customized implementation of the *SbD* paradigm. Accordingly, the main advancements with respect to the state-of-the-art [20] include (*i*) the exploitation of an aperture feeding mechanism instead of a probe feeding to significantly widen the impedance bandwidth, mitigate spurious radiation, and enable an easier manufacturing, (*ii*) the formulation of the design problem as a global optimization one rather than a parametric *trial-and-error* approach, enabling to better control the *AR* and the *XPD* in the complete radiating semi-sphere, (*iii*) the study over a wide-band of the radiation features of the resulting planar array, as well as (*iv*) the fabrication tolerance analysis on both single element and array performance.

The numerical results, concerned with the representative design of a *WWCS* radiator working at the central frequency of $f_{0}=2.45$ [GHz], have demonstrated that the proposed radiating structure provides

1. wide-band fractional impedance bandwidth (FBW≃15%), which is 12.5 times larger than that in state-of-the-art solutions based on similar *EM* mechanisms [20];
2. wide-beam radiation pattern with $HPBW\left(f_{0}\right)≃180$ [deg] in all elevation planes (versus $HPBW\left(f_{0}\right)=131$ [deg] of [20]);
3. high polarization purity with broadside $AR\left(f_{0}\right)=3.2$ [dB] and $XPD\left(f_{0}\right)=15$ [dB], together with a 3 [dB] *AR* bandwidth 12 times larger than [20].

As for the arising *WASA*, the numerical assessment has pointed out the potential of the proposed layout of the elementary radiator for the realization of wide-band circularly-polarized *WASA*s. Finally, the reliability and robustness on the fabrication tolerances of the proposed antenna layout have been verified for both the stand-alone and the array arrangement.

Furthermore, the experimental assessment of a *PCB*-manufactured prototype has verified the *FW*-simulated outcomes, confirming both the wide-band and the wide-beam features of the designed *WWCS* radiator (Figure 20 and Figure 21).

It should be pointed out that the proposed design concept and methodology are general since they can be applied to synthesize wide-band wide-beam *CP* radiators working in different operative bands. Indeed, the designer is given the freedom to choose the materials of the different layers as well as the desired target performance (i.e., bandwidth, *HPBW*, *AR*, and *XPD*) for the specific applicative scenario at hand.

Future works, beyond the scope of the current manuscript, will be aimed at assessing the possibility to exploit the stripline technology to feed the antenna and at investigating the resulting advantages and drawbacks.

## Figures and Tables

**Table 1:** Comparison in terms of central frequency ($f_{0}$), fractional bandwidth (*FBW*), polarization, elevation *HPBW* at the central frequency, and overall size (in wavelengths at $f_{0}$, $\lambda_{0}$), between the proposed *WWCS* antenna and wide-beam designs recently appeared in the scientific literature.

Ref.	Technology	$f_{0}$ [GHz]	FBW (%)	Pol.	*HPBW* [deg]	Size [$\lambda_{0}$]
[11]	Magnetoelectric dipole with parasitic patches and metallic vias	9.0	40	*LP*	156÷360	0.45×0.27×0.11
[19]	Probe-fed circular patch with rectangular parasitic patches	4.46	0.9	*LP*	156	0.84×0.84×0.03
[20]	Probe-fed circular patch with stubs and parasitic ring	2.49	1.2	*CP*	131	0.56×0.56×0.11
[21]	Comb-slot-loaded patch	8.25÷11.5	7.6÷9.1	*LP*	83÷103	0.55×0.55×0.16
[22]	Probe-fed u-slotted patch with electric walls	3.5	39	*LP*	112÷174	0.35×0.35×0.21
[23]	Aperture-fed patch with dielectric sheet and electric walls	3.5	19.7	*LP*	158÷240	0.40×0.40×0.21
[24]	Magnetic dipole antenna	24.0	2.5	*LP*	90÷140	0.57×0.57×0.06
[25]	Magnetoelectric dipole with meta-columns loading	4.15	51	*CP*	108	0.83×0.83×0.18
[26]	Crossed-printed dipoles	1.18÷1.58	3.7÷14.8	*CP*	90	0.33×0.33×0.21
This work	Aperture-fed circular patch with parasitic ring	2.45	15	*CP*	176÷189	0.50×0.50×0.59

**Table 2:** *Numerical assessment* ($\left[f_{\min},\;f_{\max}\right]=\left[2.3,\;2.6\right]$ [GHz])—*SbD*-optimized geometric descriptors of the designed *WWCS* antenna.

Parameter	Optimal Value [mm]
$R_{p}$	14.92
$L_{f}$	12.79
$L_{1}$	14.09
$W_{1}$	5.29
$L_{2}$	21.40
$W_{2}$	4.48
*D*	61.35
$R_{r}$	17.96
$W_{r}$	1.23

## Data Availability

Not applicable.
